# Supplementary material for: A natural history model for planning prostate cancer testing: Calibration and validation using Swedish registry data
Source: PLoS One. 2019 Feb 14;14(2):e0211918. doi: 10.1371/journal.pone.0211918 (PMC6375591; doi:10.1371/journal.pone.0211918)
Supplement: S1 Appendix — It also holds a comparison of survival from diagnosis by Gleason with the FHCRC model. Finally, it also includes further validation of the model. (PDF) [file pone.0211918.s001.pdf]

S1 Appendix

A natural history model for planning prostate cancer testing: calibration and validation using Swedish registry data

A Cancer onset

The cumulative probability of cancer onset was expressed as $R_o(t) = 1 - \exp\left(-\frac{\gamma_o}{2}(t-35)^2\right)$, where $\hat{\gamma}_0 = 0.0005$. This was shared with the FHCRC model which relied on the unique PCPT data set, were healthy men were biopsied. The cumulative probability of cancer onset, starting at age 35, is shown in Fig A.

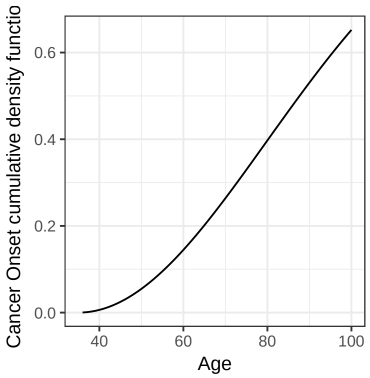

Fig A: Cumulative density of cancer onset in the natural history model.

B Swedish inputs

A number of Swedish data sources were used for extending, calibrating and validating the Stockholm Prostata model; these data sources are summarised in Table A. An important and unique data source was the SPBR register for PSA testing and prostate biopsies in the Stockholm county, which includes virtually all men having a PSA test within Stockholm county. Treatment

patterns were extracted by age, Gleason score and treatment modality for the period 2008–2013. The treatment patterns for conservative management, including active surveillance and watchful waiting, radical prostatectomy and radiotherapy, were used in the treatment sub-model.

Table A: Swedish health and population databases used for model inputs, calibration and validation.

**Registry name**	**Description**	**Study size**
Stockholm PSA and Biopsy Register	Population of men with a PSA test in Stockholm (2003–2014). Linked with registrations, deaths, migration and hospitalisations.	450,000 men
PCBaSe research database	Survival at 10 & 15 years by PSA, grade and stage for Sweden (1998–2014). PCBaSe links the National Prostate Cancer Register with deaths and migration.	80,000 cases
Life-tables	Life-tables by age and calendar year (1958–2014) from Statistics Sweden.	Sweden, Stockholm county
Prostate cancer incidence	Prostate cancer incidence by age and calendar year (1958–2014) from Socialstyrelsen.	Sweden, Stockholm county
Prostate cancer mortality	Prostate cancer mortality by age and calendar year (1980–2014) from Socialstyrelsen.	Sweden, Stockholm county

Treatment sub-model. Probabilities for treatment assignment to either active surveillance, radical prostatectomy, radiotherapy or androgen deprivation therapy were assessed from the SPBR. These values were stratified by five year age groups and Gleason score.

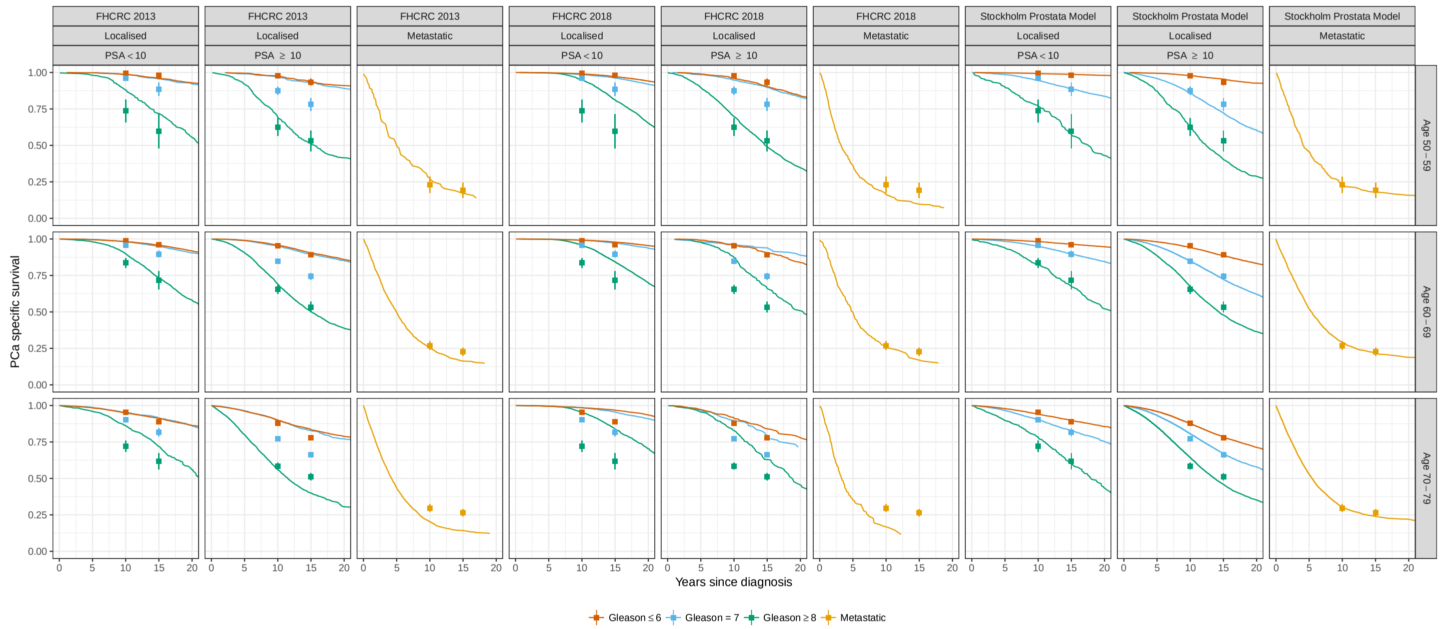

Fig B: Simulated survival from diagnosis comparing the FHCRC model in 2013 and 2018 with the Stockholm Prostata model. Overlayed together with the observed 10- and 15-year survival from PCBaSe to which the Stockholm Prostata model was fitted. Survival is stratified by age at diagnosis, PSA at diagnosis, Gleason score and cancer extent.

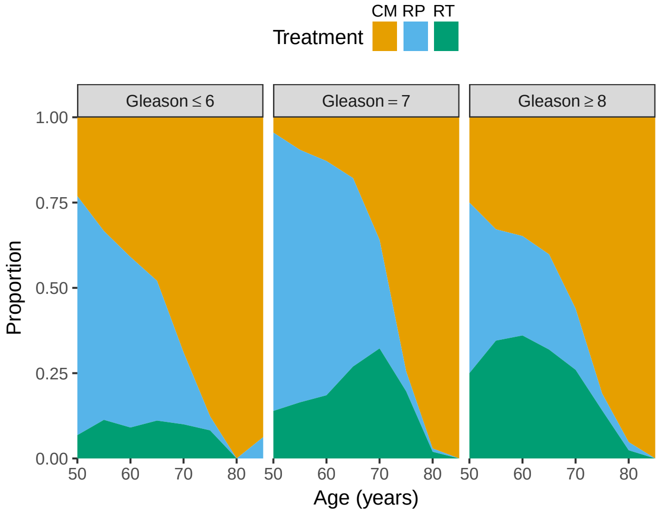

Fig C: Swedish treatment patterns from the Stockholm PSA and Biopsy Register. Treatment is divided into conservative management (CM), including active surveillance and watchful waiting, radical prostatectomy (RP) and radiotherapy (RT). The small proportion of men who fail first line therapy and subsequently have androgen deprivation therapy is indirectly modelled through palliative treatment.

The SPBR register includes virtually all men having a PSA test or prostate biopsy within the Stockholm region. Treatment data were extracted by age, Gleason grade and treatment modality for the period 2008–2013. From Fig C, the proportion of radiation therapy (RT) increases with Gleason score, radical prostatectomy (RP) is most common for Gleason 7 and decreases with age and conservative management (CM) increases with age.

We allowed for secular changes in treatment assignment. Using data for Sweden, we calculated the proportion of men diagnosed with prostate cancer diagnoses who had a radical prostatectomy (RP) within 180 days of their diagnosis; these calculations were restricted to men aged 50-69 years at diagnosis. Taking 2008 as the reference year, we calculated the odds ratio of having an RP within 180 days for the period 1988-2008 (OR=1 in 2008). We then adjusted the treatment assignment, assuming the same odds ratio for RP and radiotherapy (RT). Mathematically,

$P(\text{Treatment}j|v) = \frac{\exp(\beta_j+\phi_v)}{1+\exp(\beta_{RP}+\phi_v)+\exp(\beta_{RT}+\phi_v)}$, where β_{RP} and β_{RT} are the intercepts for 2008, $\beta_{CM} = 0$ and ϕ_v is the log odds ratio for the calendar year v.

Table B: Biopsy compliance observed in the SBPR register and used when modelling incidence from simulated currently observed PSA uptake pattern (%)

	$3 \leq$ PSA < 5	$5 \leq$ PSA < 10	PSA ≥ 10
Age < 50	37.6	56.8	77.3
$50 \leq$ Age < 60	31.1	57.3	75.4
$60 \leq$ Age < 70	23.9	48.1	69.3
$70 \leq$ Age < 80	17.5	36.9	56.0
Age ≥ 80	16.3	27.0	50.1

C Additional validation

In Fig D, we compared the simulated prostate cancer incidence with that of the Swedish and Stockholm population before the introduction of PSA testing. There is evidence for a very good fit, particularly during the potential screening ages.

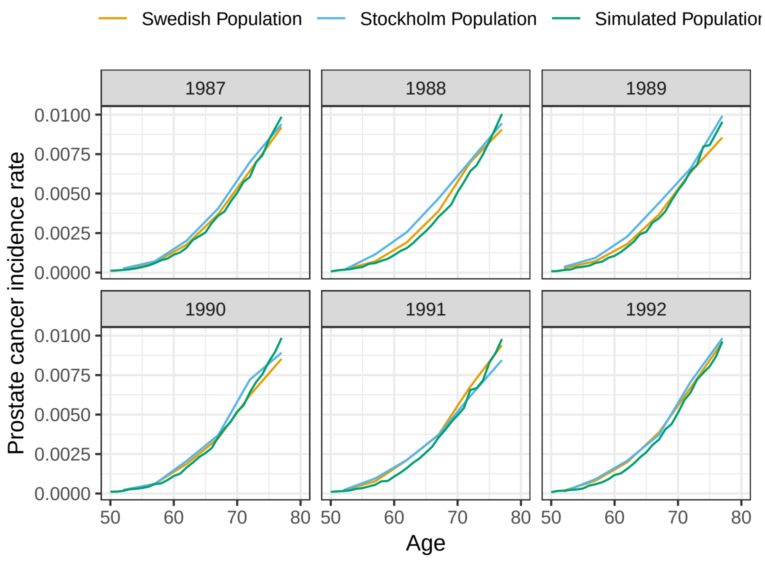

Fig D: Validating simulated symptomatic prostate cancer incidence before the introduction of PSA testing against that in the Swedish and Stockholm population.

In Fig E, we compared the simulated prostate cancer incidence with

observed rates for Sweden and Stockholm. For the earlier years, the incidence rates were underestimated in the younger ages and overestimated in the older age groups. The predictions represented the observed data well for 2016. The poorer fit is possibly an indication that the PSA sub-model is not accurately modelled for the earlier years.

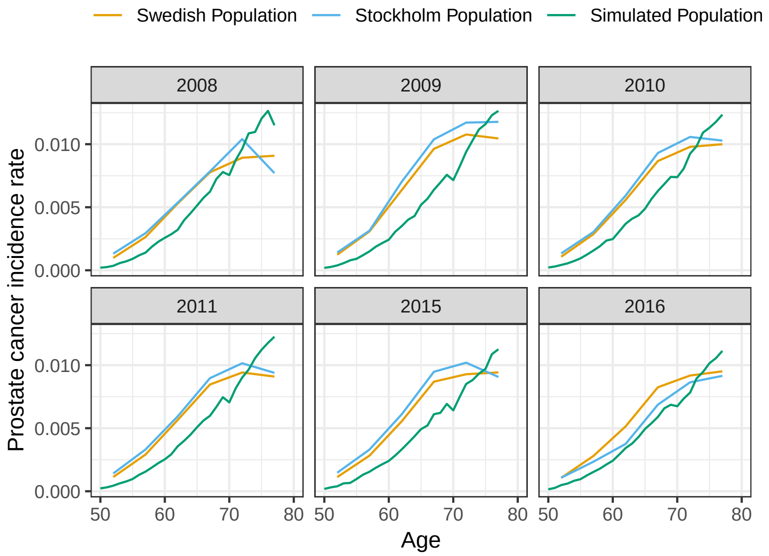

Fig E: Validating simulated prostate cancer incidence against that in the Swedish and Stockholm population.

We also validated the model comparing simulated and observed age-standardised prostate cancer mortality rates for the period 1985–2016 (Fig F). The model slightly underestimates the Swedish population mortality but seem to perform better for the latter years, particularly compared to the Stockholm data.

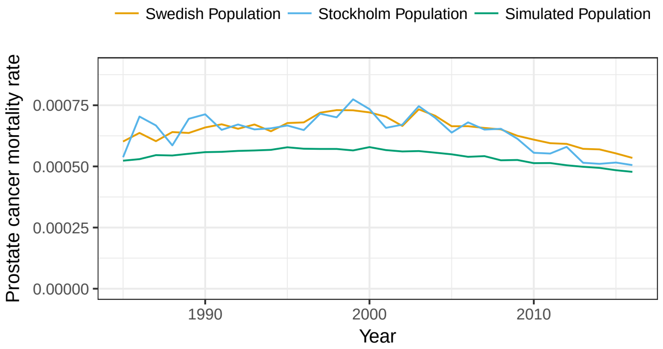

Fig F: Age-standardised prostate cancer specific mortality rates from the simulation model compared with those observed in Stockholm and Sweden.

Following the validation with age-standardised prostate cancer specific mortality rate, we validated the model over ages against the mortality rate observed in Sweden and Stockholm after the introduction of PSA testing (Fig G). The predicted prostate cancer mortality is slightly low for the earlier calendar periods but overall validates well over ages.

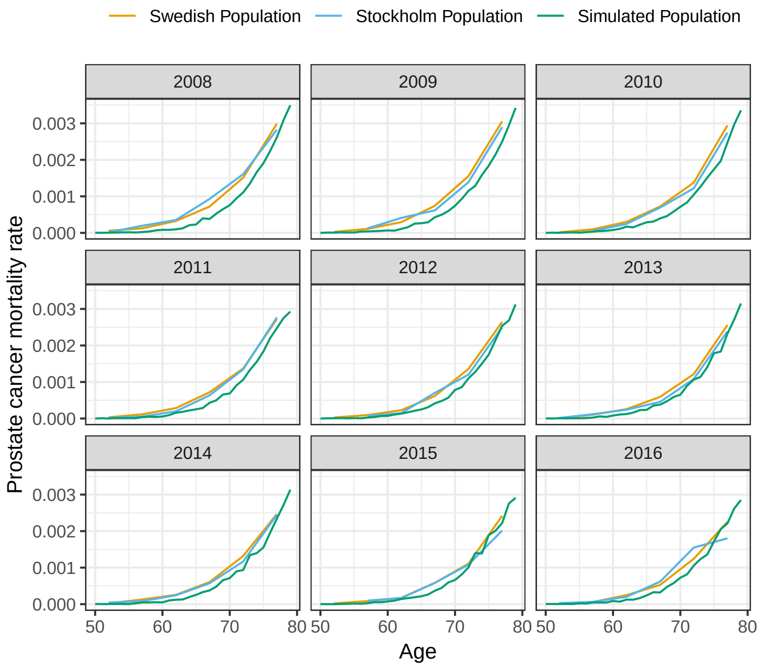

Fig G: Validating simulated prostate cancer specific mortality against that in the Swedish and Stockholm population.

Finally, in order to validate the competing risk component of the model we compared all-cause mortality rates over ages and calender period of the simulated current testing with that observed in the Swedish and Stockholm population. As can be observed in Fig H the model reflects the all-cause mortality well over ages and calendar periods.

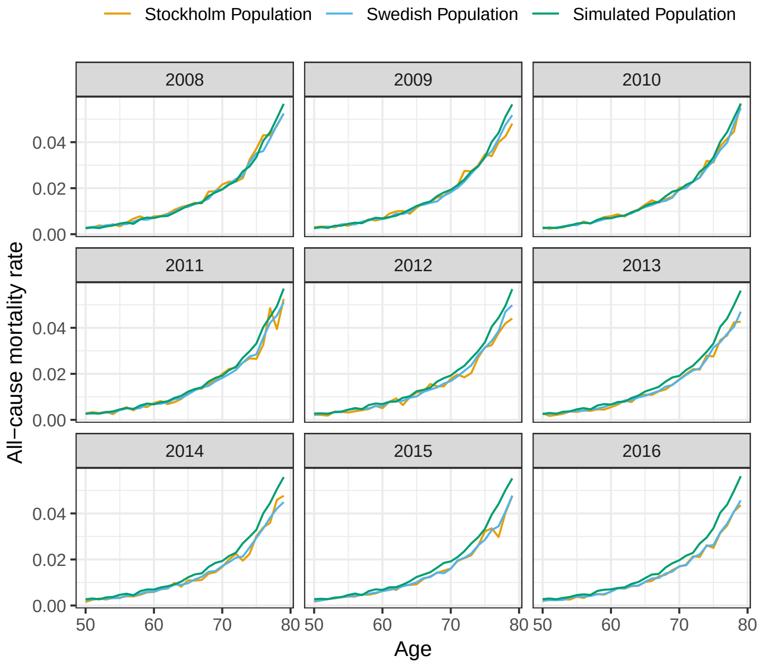

Fig H: Validating simulated all-cause mortality against that in the Swedish and Stockholm population.
